# Kidney-sparing resection of concomitant renal artery and renal vein aneurysms by single-port retroperitoneoscopic surgery: a case report

**DOI:** 10.3389/fmed.2026.1912155

**Published:** 2026-08-14

**Authors:** Chi Gan, Xiyi Wei, Da Zhong, Xiao Li, Jie Yang, Ninghong Song

**Affiliations:** 1 Department of Urologic Surgery, Jiangsu Cancer Hospital and Jiangsu Institute of Cancer Research and Affiliated Cancer Hospital of Nanjing Medical University, Nanjing, China; 2 Department of Urology, The First Affiliated Hospital with Nanjing Medical University, Nanjing, China

**Keywords:** kidney-sparing surgery, nephron-sparing surgery, renal artery aneurysm, renal vein aneurysm, retroperitoneoscopy, single-port laparoscopy

## Abstract

Renal artery aneurysm and renal vein aneurysm are rare vascular lesions, and their coexistence within the same kidney poses considerable diagnostic and surgical challenges. We report a 51-year-old woman presenting with recurrent left flank and back pain. Computed tomography angiography demonstrated a 2.1-cm upper-pole renal artery aneurysm arising from a terminal intrarenal arterial branch and a 3.0-cm renal vein aneurysm arising from a large intrarenal venous tributary in the mid-pole renal sinus. Nephrectomy had been recommended at another institution because of the anatomical complexity, but the patient strongly wished to preserve the affected kidney. She underwent single-port retroperitoneoscopic excision of both aneurysms, with closure of the terminal arterial stump, primary closure of the venous wall defect, and renorrhaphy. The operative time was 95 min, warm ischemia time was 20 min, and estimated blood loss was 30 mL. Histopathological examination confirmed the diagnoses. At 4 months after surgery, color Doppler ultrasonography demonstrated preserved main left renal arterial flow and intrarenal arterial perfusion without Doppler evidence of hemodynamically significant main renal artery stenosis. Dedicated renal venous Doppler assessment and differential renal function testing were not performed. This case demonstrates that single-port retroperitoneoscopic kidney-sparing excision can be performed in selected patients with concomitant intrarenal arterial and venous aneurysms.

## Introduction

Renal vascular aneurysms represent an unusual group of lesions that may be discovered incidentally or present with pain, hematuria, hypertension, or other complications (1–3). When such lesions are centrally located and involve both the arterial and venous systems of the same kidney, surgical management becomes particularly challenging because treatment must balance complete lesion excision against preservation of renal function (3–6).

In complex cases, nephrectomy may be considered because of technical difficulty and concern regarding bleeding or vascular injury (7). However, for selected patients, kidney-sparing surgery may provide an effective alternative when meticulous preoperative imaging and careful operative planning are available (4–6).

Here, we report a 51-year-old woman presenting with recurrent left flank and back pain in the setting of concomitant intrarenal renal artery and renal vein aneurysms, who underwent single-port retroperitoneoscopic kidney-sparing excision of both lesions.

## Case description

A 51-year-old woman was referred to our institution with recurrent left flank and back pain. She had initially been evaluated at a local hospital, where computed tomography revealed a 2.1 cm renal artery aneurysm and a 3.0 cm renal vein aneurysm in the left kidney. Owing to the complexity of the lesions and the expected difficulty of renal preservation, particularly in view of the large centrally located venous aneurysm, nephrectomy had been recommended at the outside hospital. The patient, however, strongly wished to preserve the affected kidney and therefore sought further treatment at our center.

She was admitted on February 18, 2025. Her past medical history was unremarkable. She denied gross hematuria, fever, nausea, and vomiting. She had no history of smoking or alcohol consumption, and there was no relevant family history. On admission, her blood pressure was 132/86 mmHg, heart rate was 86 beats per minute, and body temperature was 37.0 °C. Routine laboratory investigations were within normal limits, and baseline renal function was preserved, with a blood urea level of 4.6 mmol/L, serum creatinine level of 53 μmol/L, and uric acid level of 220 μmol/L.

**Figure 1:**
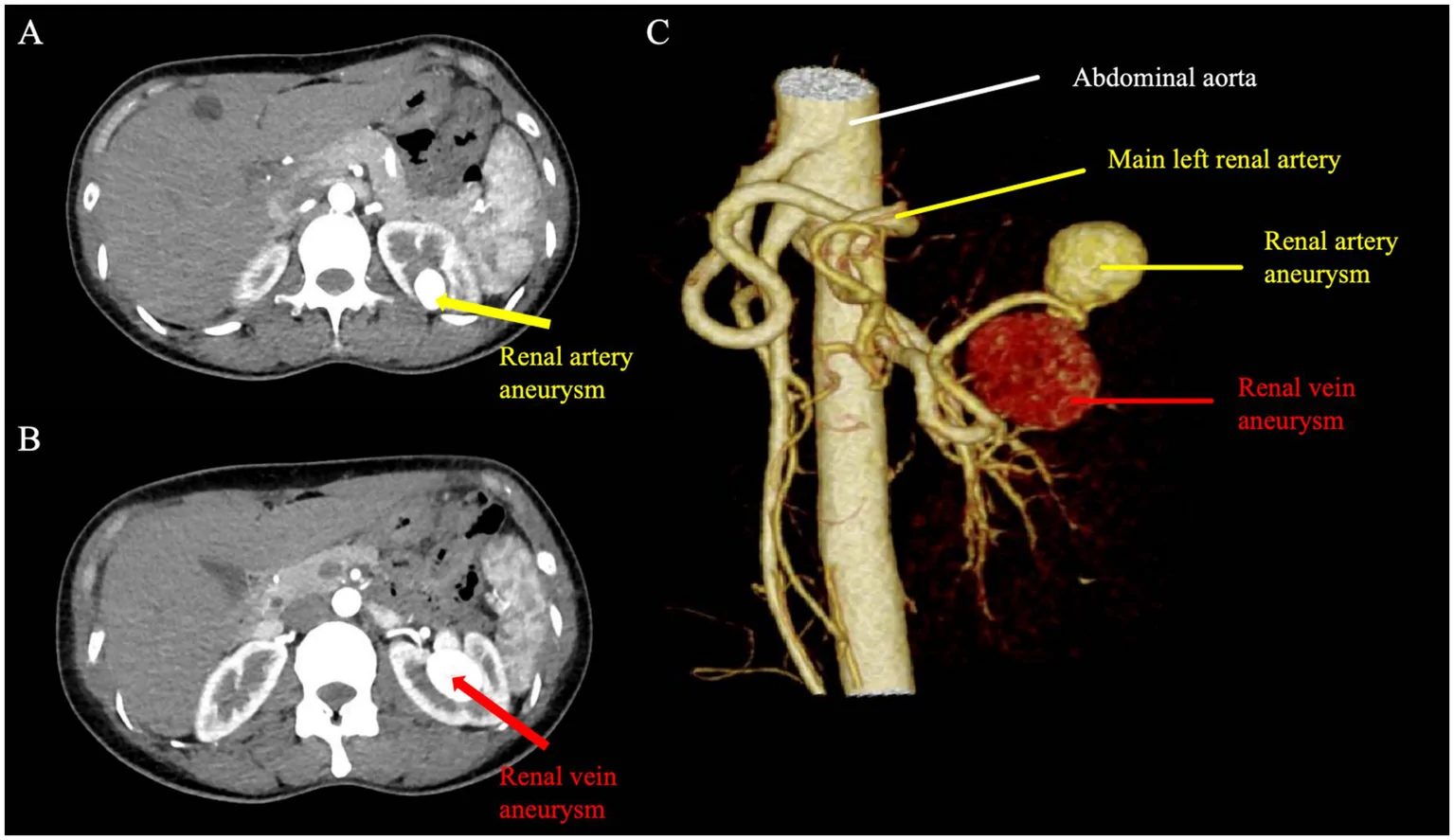
Preoperative computed tomography angiography and three-dimensional vascular reconstruction of concomitant renal artery and renal vein aneurysms. **(A)** Axial arterial-phase computed tomography angiography demonstrating a left renal artery aneurysm located in the upper pole of the left kidney. The aneurysm measured approximately 23.0 mm in maximum diameter. **(B)** Axial venous-phase computed tomography angiography showing a left renal vein aneurysm located in the renal sinus region of the mid-pole of the left kidney. The aneurysm measured approximately 32.2 mm in maximum diameter. **(C)** Three-dimensional vascular reconstruction demonstrating the spatial relationship between the abdominal aorta, main left renal artery, renal artery aneurysm, and renal vein aneurysm. The renal artery aneurysm and renal vein aneurysm were located separately within the same kidney, with no evidence of direct arteriovenous communication.

Computed tomography angiography demonstrated a 21 × 15 × 17-mm nodular enhancing lesion in the upper pole of the left kidney. The lesion showed homogeneous arterial-phase enhancement similar to that of the renal artery and arose from a terminal intrarenal arterial branch supplying the upper pole (Figure 1A). No substantial distal arterial continuation was present beyond the aneurysm. A second nodular enhancing lesion measuring 30 × 22 × 30 mm was located in the mid-pole renal sinus adjacent to the renal pelvis. The lesion showed lower enhancement than the renal artery during the arterial phase and filled during the venous phase. It arose from a large intrarenal venous tributary that drained into the main left renal vein. The venous aneurysm did not originate from the extrarenal main left renal vein and communicated with its parent venous tributary through a broad base (Figure 1B). Three-dimensional CTA reconstruction demonstrated the spatial relationship between the renal artery aneurysm and renal vein aneurysm, as well as their separate arterial and venous origins. No direct arteriovenous fistulous communication was present between the lesions (Figure 1C). No obvious arterial wall thickening, calcification, or luminal stenosis was identified.

Based on the imaging findings, the patient was diagnosed with concomitant left intrarenal arterial and venous aneurysms. The available management strategies, including imaging surveillance, endovascular treatment, open or minimally invasive *in situ* surgery, ex vivo repair with renal autotransplantation, and nephrectomy, were discussed with the patient. The decision to intervene was based on her recurrent clinical presentation, the concomitant 3.0-cm centrally located venous aneurysm, the coexistence of two vascular lesions within the same kidney, and her strong preference for renal preservation. Because both lesions were focal and accessible through the posterior renal surface, single-port retroperitoneoscopic kidney-sparing excision was selected.

On February 19, 2025, the patient underwent single-port retroperitoneoscopic surgery under general anesthesia in the right lateral decubitus position. A 1.5-cm incision was made approximately 2 cm above the iliac crest. The muscle layers were bluntly separated to enter the retroperitoneal space, which was developed using an 800-mL balloon dilator. A disposable multichannel single-port trocar was inserted, and pneumoretroperitoneum was established.

After dissection of the extraperitoneal fat, the renal hilum was approached along the anterior border of the psoas muscle. The left renal artery was isolated at the renal hilum and temporarily clamped. Only the renal artery was clamped; the main left renal vein was not clamped, and en bloc hilar clamping was not performed.

**Figure 2:**
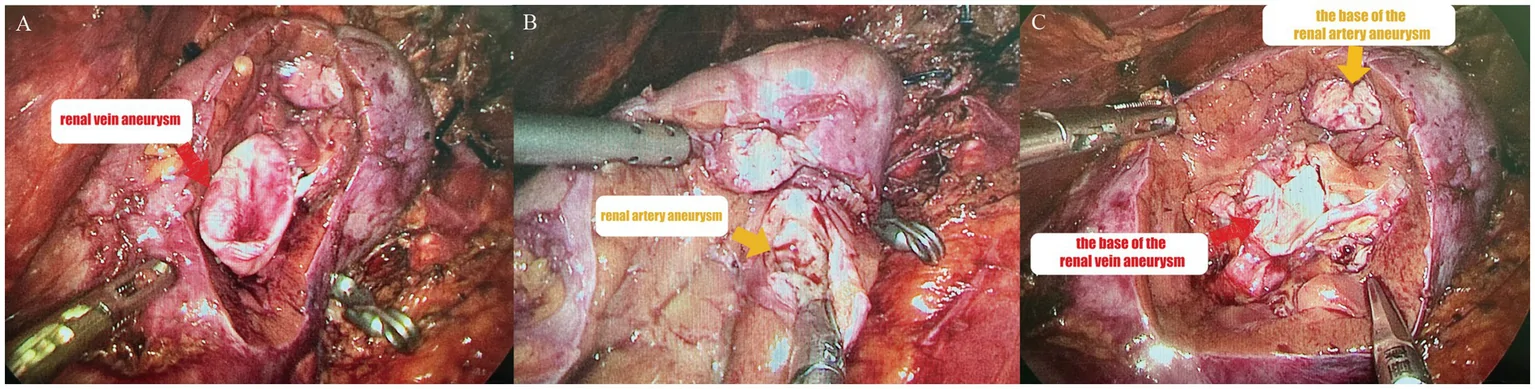
Intraoperative findings during single-port retroperitoneoscopic kidney-sparing surgery. **(A)** Intraoperative exposure of the renal artery aneurysm located at the upper pole of the left kidney. **(B)** Intraoperative identification of the centrally located renal vein aneurysm after extension of the parenchymal incision toward the mid-pole near the renal pelvis. **(C)** Intraoperative view of the bases of the renal artery aneurysm and renal vein aneurysm after aneurysm dissection.

Gerota’s fascia was opened, and the kidney was mobilized. The approximately 2-cm arterial aneurysm was identified within the upper-pole renal parenchyma (Figure 2A). The aneurysm arose from a terminal intrarenal arterial branch supplying the upper pole. The posterior renal parenchyma was incised along the margin of the aneurysm, and the lesion was dissected to its base. The aneurysm was excised together with its terminal feeding branch, and the arterial stump was closed with a running absorbable suture. No arterial anastomosis, reimplantation, patch angioplasty, or interposition graft was performed.

The renal parenchymal incision was extended toward the mid-pole adjacent to the renal pelvis. The approximately 3-cm venous aneurysm was identified within the renal sinus (Figure 2B). The aneurysm arose from a large intrarenal venous tributary draining into the main left renal vein and communicated with the parent venous tributary through a broad base. Both aneurysms were dissected to their respective bases, exposing the terminal feeding arterial branch and the broad communicating base of the venous aneurysm (Figure 2C). Interruption of renal arterial inflow reduced renal venous return and created a low-flow operative field. The venous aneurysm was excised along its communicating base without clamping the main renal vein, and the resulting venous wall defect was closed primarily with a running absorbable suture.

**Figure 3:**
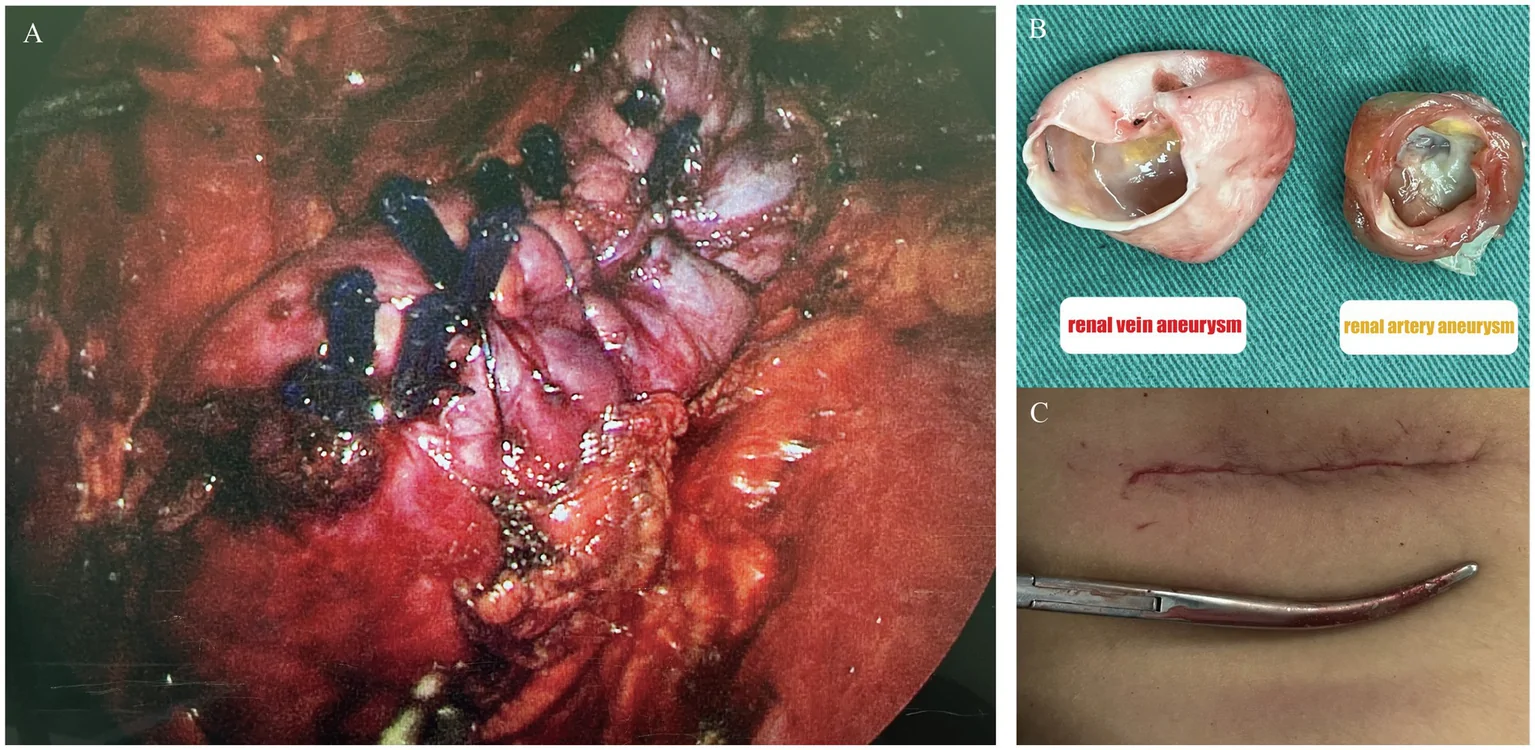
Reconstruction, gross appearance of the resected lesions, and postoperative single-port incision. **(A)** Renorrhaphy and closure of the resection bed after excision of both aneurysms. **(B)** Gross specimen showing the resected renal artery aneurysm and renal vein aneurysm. **(C)** Appearance of the single-port incision after wound closure at the end of the procedure.

After separate closure of the arterial stump and venous defect, continuous renorrhaphy was performed using a 1–0 absorbable suture (Figure 3A). The renal arterial clamp was released after 20 min of warm ischemia. The preserved renal parenchyma regained a uniform pink color and normal turgor. No focal ischemic discoloration, renal swelling, venous congestion, or active bleeding from the arterial stump, venous closure site, or renal parenchymal margin was observed. No intraoperative Doppler ultrasonography, indocyanine green fluorescence angiography, or intraoperative angiography was used.

Hemostatic material was applied, and the aneurysm specimens were retrieved in a specimen bag. The resected renal artery aneurysm and renal vein aneurysm were retrieved intact and are shown in Figure 3B. The retroperitoneal operative field was irrigated, hemostasis was confirmed, and the single-port incision was closed (Figure 3C).

The procedure consisted of excision of the renal artery aneurysm with closure of the terminal arterial stump, excision of the renal vein aneurysm with primary closure of the venous wall defect, and renorrhaphy. The operation was completed without conversion to multi-port or open surgery. Total operative time was 95 min and estimated blood loss was 30 mL.

Postoperative recovery was uneventful. Serum creatinine increased from 53 μmol/L preoperatively to 74.3 μmol/L postoperatively, without clinically significant impairment of overall renal function. The patient was discharged on February 26, 2025.

**Figure 4:**
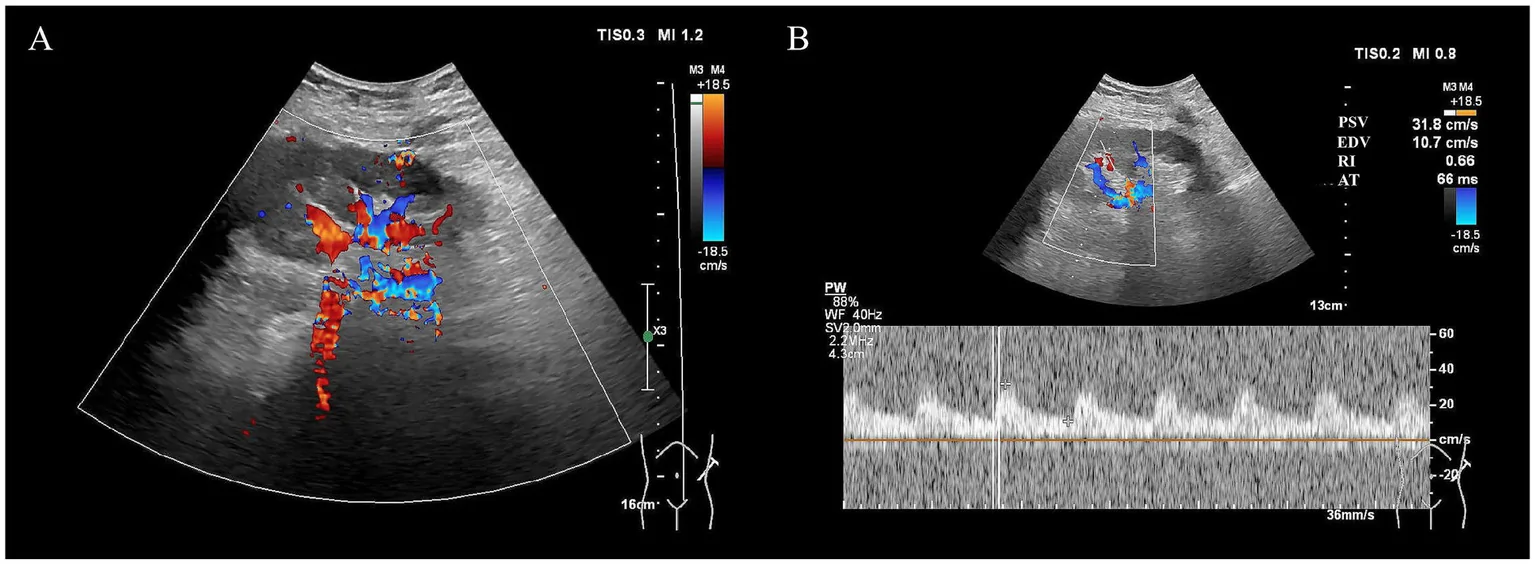
Color Doppler ultrasonography of the operated left kidney at 4 months after surgery **(A)** Color Doppler image demonstrating preservation of the left kidney and visualization of the intrarenal arterial tree **(B)** Representative spectral Doppler waveform of a left interlobar artery, demonstrating preserved intrarenal arterial flow.

At 4 months after surgery, the patient remained free of recurrent flank or back pain and reported no procedure-related symptoms. Color Doppler ultrasonography demonstrated preservation of the operated left kidney and clear visualization of the intrarenal arterial tree (Figure 4A). The operated left kidney measured 110 mm in longitudinal length, without obvious renal atrophy. The main left renal artery was clearly visualized, with a diameter of 6.0 mm and a peak systolic velocity of 102 cm/s. No abnormal color aliasing or turbulent flow was observed. A representative left interlobar arterial waveform showed a peak systolic velocity of approximately 32 cm/s and an acceleration time of 66 ms (Figure 4B). These findings demonstrated preserved main left renal arterial flow and intrarenal arterial perfusion without Doppler evidence of hemodynamically significant main renal artery stenosis.

Dedicated Doppler assessment of the left renal vein, postoperative CTA or MR angiography, and differential renal function testing were not performed. Therefore, postoperative renal venous patency and differential function of the operated kidney were not objectively assessed.

## Discussion

The principal novelty of this case lies in the coexistence of two anatomically distinct intrarenal vascular aneurysms within the same kidney: a terminal upper-pole arterial branch aneurysm and a separate mid-pole intrarenal venous tributary aneurysm. Multiphasic CTA demonstrated no direct arteriovenous fistulous communication between the lesions. Renal vein aneurysms are exceptionally uncommon, and the available literature consists predominantly of isolated case reports and small literature reviews (8). The simultaneous treatment of two separate intrarenal arterial and venous aneurysms through a single-port retroperitoneoscopic kidney-sparing approach further distinguishes the present case.

Management of renal vascular aneurysms should be individualized according to symptoms, aneurysm size and growth, anatomical location, branch-vessel involvement, baseline renal function, and the feasibility of preserving arterial inflow and venous drainage. Stable asymptomatic lesions may be managed with imaging surveillance. Endovascular options for renal artery aneurysms include selective coil or plug embolization, parent-branch embolization, balloon- or stent-assisted coiling, covered stent exclusion, and flow-diverting devices. Surgical alternatives include open or minimally invasive *in situ* aneurysm excision with primary closure, arteriorrhaphy, patch angioplasty, arterial reimplantation, end-to-end anastomosis, interposition grafting, or bypass. Ex vivo repair with renal autotransplantation may be used for complex hilar or multibranch lesions that cannot be safely treated in situ. Reported management strategies for renal vein aneurysms include surveillance, surgical excision with venous defect closure or reconstruction, and selective endovascular occlusion. Nephrectomy may be required when safe vascular control and renal preservation are not technically feasible (9–12).

In this patient, the 2.1-cm renal artery aneurysm alone was not the sole indication for intervention. Current recommendations emphasize that treatment decisions should incorporate symptoms, aneurysm anatomy, growth, and patient-specific factors rather than aneurysm diameter alone (4). The decision was based on the recurrent clinical presentation, concomitant 3.0-cm centrally located renal vein aneurysm, coexistence of two vascular lesions within the same kidney, and the patient’s strong preference for renal preservation. Although a direct causal relationship between the nonspecific flank pain and the aneurysms could not be established, definitive treatment was selected after shared decision-making (4).

Endovascular treatment was not selected because the arterial aneurysm arose from a small terminal intrarenal branch without an adequate landing zone for covered stent placement. Embolization would have sacrificed the corresponding upper-pole arterial territory and increased the risk of segmental renal infarction (10, 11). For the centrally located venous aneurysm, complete endovascular exclusion would have compromised the associated intrarenal venous drainage and introduced risks of thrombosis or venous outflow obstruction. Published endovascular treatment of renal vein aneurysms remains limited to highly selected anatomical settings. Open *in situ* surgery or ex vivo repair with renal autotransplantation would have provided wider exposure but were more invasive than required because both lesions were focal and accessible in situ (12).

The retroperitoneal route provided direct access to the renal hilum and posterior kidney, permitted early control of renal arterial inflow, avoided transperitoneal mobilization of the bowel, spleen, and pancreas, and allowed both aneurysms to be treated within the same operative field. The single-port technique was selected on the basis of the operating team’s experience and its ability to limit the abdominal wall incision. However, instrument crowding, reduced triangulation, and limited maneuverability increase the difficulty of vascular exposure, suturing, and emergency hemorrhage control. The present case does not establish superiority over conventional multi-port, robotic, or open surgery; it demonstrates the feasibility of single-port retroperitoneoscopic treatment in a selected patient at an experienced center (13, 14).

Direct *in situ* excision allowed simultaneous treatment of both lesions, avoided nephrectomy, preserved most renal parenchyma, and provided complete specimens for histopathological examination. Potential complications included major hemorrhage, warm ischemic injury, segmental renal infarction following division of the terminal feeding artery, arterial stump bleeding or pseudoaneurysm formation, venous stenosis or thrombosis, impaired venous outflow, renal congestion, and the need for conversion or rescue nephrectomy (5, 11). No intraoperative Doppler ultrasonography or indocyanine green fluorescence angiography was used. Therefore, intraoperative assessment was limited to macroscopic reperfusion and confirmation of hemostasis rather than objective small-vessel flow imaging. Indocyanine green fluorescence angiography has been used to evaluate renal arterial flow and renal parenchymal perfusion during renal artery aneurysm surgery (15).

The pathogenesis of concomitant renal arterial and venous aneurysms remains uncertain. Renal artery aneurysms may be associated with congenital or acquired structural abnormalities of the arterial wall (4, 9). Primary renal vein aneurysms have been attributed to congenital structural weakness of the venous wall, whereas secondary venous dilatation may occur in association with abnormal venous anatomy or arteriovenous shunting. In this patient, no direct arteriovenous fistula was present. Both lesions showed fibrous wall hyperplasia, hyaline degeneration, and myxoid degeneration. These similar but nonspecific findings support a shared degenerative or dysplastic vascular-wall abnormality. However, no specific systemic vasculopathy was established, and local hemodynamic factors or a coincidental association cannot be excluded.

At 4 months after surgery, color Doppler ultrasonography demonstrated preserved main left renal arterial flow and intrarenal arterial perfusion. The operated left kidney showed no obvious atrophy. Nevertheless, dedicated postoperative renal venous imaging and differential renal function testing were not performed. Serum creatinine was therefore interpreted only as an indicator of overall bilateral renal function. Long-term surveillance should assess arterial stump pseudoaneurysm, arterial stenosis or thrombosis, segmental renal infarction, venous thrombosis or outflow obstruction, renal congestion, renal atrophy, recurrent aneurysm, recurrent flank pain or hematuria, and new or worsening hypertension (11).

This report has several limitations. It describes a single patient, objective intraoperative perfusion imaging was not used, postoperative renal venous patency and differential renal function were not assessed, representative microscopic images were unavailable, and follow-up was limited to 4 months. Therefore, the wider applicability and long-term durability of this approach remain uncertain.

## Conclusion

Concomitant intrarenal renal artery and renal vein aneurysms are rare and technically challenging. In this patient, single-port retroperitoneoscopic excision of both lesions with closure of the terminal arterial stump and primary closure of the venous wall defect avoided nephrectomy and achieved a favorable short-term clinical outcome. Postoperative Doppler ultrasonography demonstrated preserved main renal arterial flow and intrarenal arterial perfusion. Renal venous patency and differential renal function were not objectively assessed. This approach can be performed in selected patients when detailed preoperative vascular assessment, reliable hilar control, and sufficient minimally invasive surgical experience are available.

## Data Availability

The original contributions presented in the study are included in the article/supplementary material, further inquiries can be directed to the corresponding authors.
